# Membrane Heterogeneity Controls Cellular Endocytic Trafficking

**DOI:** 10.3389/fcell.2020.00757

**Published:** 2020-08-05

**Authors:** Gregory M. I. Redpath, Verena M. Betzler, Pascal Rossatti, Jérémie Rossy

**Affiliations:** ^1^Department of Biochemistry, School of Biomedical Sciences, University of Otago, Dunedin, New Zealand; ^2^The ANZAC Research Institute, Concord Repatriation General Hospital, Concord, NSW, Australia; ^3^Graduate School for Cellular and Biomedical Sciences, University of Bern, Bern, Switzerland; ^4^Biotechnology Institute Thurgau (BITg) at the University of Konstanz, Kreuzlingen, Switzerland; ^5^Department of Biology, University of Konstanz, Konstanz, Germany

**Keywords:** endosomal sorting, Rab11, retromer/retriever, clathrin, endophilin, CLIC/GEEC, phosphoinositide, phosphatidylserine

## Abstract

Endocytic trafficking relies on highly localized events in cell membranes. Endocytosis involves the gathering of protein (cargo/receptor) at distinct plasma membrane locations defined by specific lipid and protein compositions. Simultaneously, the molecular machinery that drives invagination and eventually scission of the endocytic vesicle assembles at the very same place on the inner leaflet of the membrane. It is membrane heterogeneity – the existence of specific lipid and protein domains in localized regions of membranes – that creates the distinct molecular identity required for an endocytic event to occur precisely when and where it is required rather than at some random location within the plasma membrane. Accumulating evidence leads us to believe that the trafficking fate of internalized proteins is sealed following endocytosis, as this distinct membrane identity is preserved through the endocytic pathway, upon fusion of endocytic vesicles with early and sorting endosomes. In fact, just like at the plasma membrane, multiple domains coexist at the surface of these endosomes, regulating local membrane tubulation, fission and sorting to recycling pathways or to the *trans*-Golgi network via late endosomes. From here, membrane heterogeneity ensures that fusion events between intracellular vesicles and larger compartments are spatially regulated to promote the transport of cargoes to their intracellular destination.

## Introduction

In most situations, cells respond to extracellular stimuli through the binding and activation of cell surface receptors to extracellular ligands. Following activation, receptors are internalized, sorted for degradation or for recycling to regulate the composition, distribution and density of the pool of available receptors at the cell surface and ultimately to modulate the signaling response (Scita and Di Fiore, 2010). While they were first regarded as being part of a constitutive process, it is now well established that the manifold mechanisms that make up cellular trafficking are highly regulated. Determination of when and where events such as packaging of cargoes or scission and fusion of vesicles is central to the regulation of endocytic trafficking and relies greatly on heterogeneity of membranes. Successive models for cell membranes – fluid mosaic (Singer and Nicolson, 1972), lipid rafts (Simons and Ikonen, 1997), picket-fence (Kusumi et al., 2011), or active composite (Rao and Mayor, 2014) tend toward a perception that membranes are not homogenous mixtures of lipids and proteins, but rather heterogeneous and compartmentalized (Jacobson et al., 2019). The interplay between different lipid species, membrane proteins, local dynamics of actin polymerisation and membrane curvature creates non-homogenous membrane domains that drive and regulate endocytosis, sorting and recycling. In this review, we highlight how at every step of the endocytic pathway, membrane heterogeneity generates specific domains that ultimately regulate cargo fate. We focus on domains constituted of lipid species – phosphoinositides, cholesterol and phosphatidylserine – which most contribute to membrane heterogeneity during intracellular trafficking, yet the concepts we refer to are likely to extend to other lipid species in similar cellular processes.

Phosphoinositides or phosphatidylserine membrane domains can be generated through clustering by Ca^2+^ (Boettcher et al., 2011; Wen et al., 2018) or by BAR domain containing proteins (Saarikangas et al., 2009; Zhao et al., 2013; Picas et al., 2014). Other mechanisms of phosphoinositide clustering are comprehensively described in an excellent recent review (Picas et al., 2016). However, in the context of endocytic trafficking, membrane heterogeneity most often results from the recruitment of membrane remodeling proteins – phospholipid-modifying enzymes, flippases, curvature-inducing proteins or regulators of actin polymerization – by coat proteins, adaptors, or lipids that already define membrane domains with distinct properties. Hence, in this review we consider that heterogenous membrane domains do not dissipate during transitions from one step of the endocytic journey to the next, but rather that endocytic transitions are driven by shedding and recruitment of membrane remodeling proteins.

Heterogeneity is one of the main factors determining where and which type of endocytosis will be triggered. Endocytic events occur in unique membrane domains which then undergo precise changes as they mature to facilitate formation of endosomes (Danson et al., 2013; Boucrot et al., 2015; Nakatsu et al., 2015; Posor et al., 2015). Membrane heterogeneity is propagated further through the endosomal pathway by phosphoinositide metabolism that switches phosphoinositide species throughout the endocytic trafficking process, ultimately regulating every step of the intracellular sorting process that occurs following endocytosis.

Below, we discuss how membrane heterogeneity generates domains that regulate the initiation and progression of clathrin-dependent endocytosis, fast endophilin-mediated endocytosis and clathrin independent carrier/glycosylphosphatidylinositol -anchored protein enriched endocytic compartments (CLIC/GEEC) endocytosis. We address the contribution of different phosphoinositide species and cholesterol, actin and membrane curvature to ensuring the mode of endocytosis remains tightly regulated. We then detail how phosphoinositide and phosphatidylserine membrane domains ensure each cargo progresses through the endosomal sorting steps to its target destination. Ultimately, we propose a model in which phosphoinositide and phosphatidylserine generate membrane heterogeneity either during or immediately following endocytosis and segregates cargoes into defined membrane domains. Therefore, our model implies that the trafficking fate of cargoes is decided at the endocytosis stage, and these domains are maintained throughout their respective sorting pathways until the required cellular destination is reached. In short, this review highlights how at every step of the endocytic pathway, membrane heterogeneity generates specific domains that ultimately regulate cargo fate.

## Endocytosis

Membrane heterogeneity stands at the center of regulating entry of receptors and cargoes into the cell *via* endocytosis. Endocytosis involves the invagination of the plasma membrane containing the cargo, followed by scission of the invaginated region from the plasma membrane, and eventually the formation of a distinct endocytic vesicle. Endocytosis is segregated into two broad modes- clathrin-dependent and clathrin-independent. Clathrin-dependent endocytosis is the best studied of the two and is defined by the presence of a clathrin coat on the endocytic vesicle. Clathrin-independent endocytosis represents multiple, less defined internalization pathways. Membrane heterogeneity contributes to the selection of the mode of internalization and ultimately regulates how, when and where a cargo enters the cell. It further ensures that clathrin-dependent and -independent endocytosis remain distinct and selective and that the many elements shared exert specific functions in each mode of endocytosis.

### Clathrin-Dependent Endocytosis

Clathrin-dependent endocytosis is defined by extrusion of the plasma membrane into an endocytic vesicle surrounded by a clathrin lattice followed by scission from the membrane. Spatial and temporal heterogeneity of phosphoinositide metabolism regulates the timing of each step of clathrin-dependent endocytosis, including clathrin coat nucleation, endocytic pit formation, vesicle scission and clathrin uncoating [extensively reviewed in Mettlen et al. (2018)].

**FIGURE 1:**
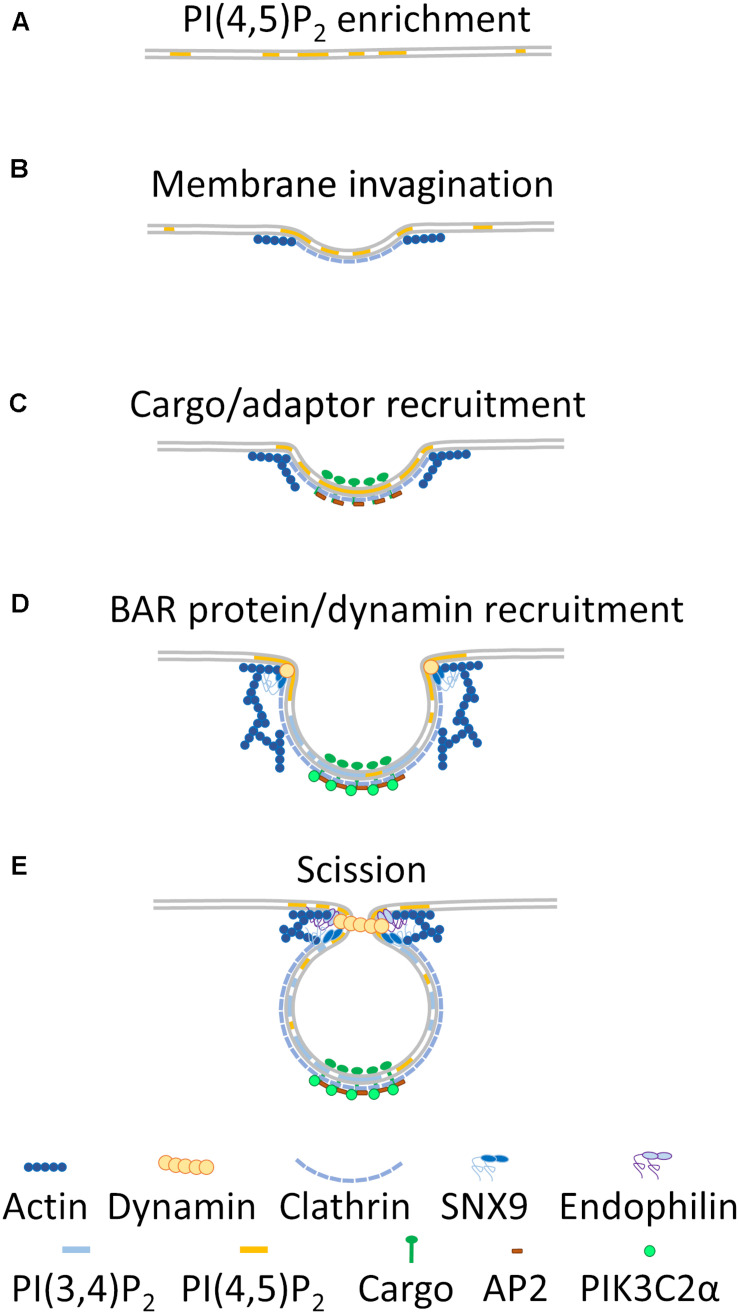
Membrane heterogeneity regulating clathrin-dependent endocytosis. In relatively flat regions of the plasma membrane, PI(4,5)P_2_ enrichment occurs **(A)**. This enrichment leads to induction of membrane curvature that recruits clathrin and branched actin to the nascent endocytic pit **(B)**. Cargo is enriched within this pit, and AP2 is recruited to the cargo and clathrin coat **(C)**. Further actin branching and extrusion of the clathrin coated pit leads to recruitment of PIK3C2α, which metabolizes PI(4,5)P_2_ to PI(3,4)P_2_ around the clathrin coat, while BAR-domain containing proteins and dynamin are recruited to the highly curved neck region of the pit **(D)**. Finally, increased recruitment of BAR-domain containing proteins, branched actin and dynamin to the neck region leads to constriction of the endosome and scission from the plasma membrane **(E)**.

Phosphoinositides are membrane lipid species that are readily modifiable by phosphorylation at the 3, 4, or 5 hydroxyl positions on the inositol ring and represent a major source of membrane heterogeneity within the cell (Schink et al., 2016). In this regard, they are especially important for intracellular trafficking (Wang et al., 2019). For clathrin-dependent endocytosis, heterogeneity mainly results from plasma membrane regions enriched in phosphoinositide 4,5 phosphate [PI(4,5)P_2_] and displaying high membrane curvature, which together provide the initial impetus for formation of a clathrin coated pit (Figure 1A). Asymmetric enrichments of PI(4,5)P_2_ lead to spontaneous induction of membrane curvature *in vitro* through a mechanism that remains to be elucidated (Shukla et al., 2019). In turn, membrane curvature leads to recruitment and stabilization of a clathrin-lattice at the plasma membrane (Figure 1B) (Jost et al., 1998; Cremona et al., 1999). This lattice is further stabilized by formation of actin filaments promoted by direct binding of actin regulatory proteins to areas enriched in PI(4,5)P_2_ in the membrane (Figure 1B) (Yin and Janmey, 2003; Leyton-Puig et al., 2017). Ligand binding promotes translocation of receptors and cargo to these stabilized clathrin plaques (Leyton-Puig et al., 2017). Localized enrichments in PI(4,5)P_2_ are therefore required to initiate clathrin-dependent endocytosis and stabilize the clathrin lattice in order to allow cargo entry into the forming endocytic pit.

Recruitment of adaptor proteins to the stabilized clathrin lattice leads to an additional local increase of PI(4,5)P_2_, further creating a membrane environment distinct from the surrounding plasma membrane (Figure 1C) (Krauss et al., 2006). The adaptor protein AP-2 interacts with PI(4,5)P_2_, which stabilizes it in an open conformation and facilitates cargo and clathrin binding at the forming lattice. Open AP-2 also recruits phosphatidylinositol kinase type-I which generates more PI(4,5)P_2_ (Krauss et al., 2006; Jackson et al., 2010). Heterogeneity in the plasma membrane resulting from phosphoinositide (PI) enrichment is therefore a crucial step for initiating and stabilizing clathrin endocytic pits.

At this stage, a clathrin pit begins to resemble a 3D endocytic structure being extruded from the plasma membrane, in which a segregation between the endocytic pit and surrounding plasma membrane is evident (Figures 1B–D). Rather than being a two-dimensional assembly of proteins on the plasma membrane, the clathrin-coated pit has a strong dimension of curvature that allows the recruitment of membrane curvature-sensing and -inducing BAR-domain containing proteins at the neck of the budding vesicles (Figure 1D) (Lo et al., 2017; Schöneberg et al., 2017). This geometric membrane heterogeneity is reinforced by PI(4,5)P_2_ concentration to the neck region of the pit and facilitates the recruitment of dynamin (Figure 1D) (Zheng et al., 1996). Dynamin ultimately exerts a constricting force on the endocytic pit which leads to scission from the plasma membrane (Ferguson et al., 2009).

While clathrin pits progress to fully formed endocytic vesicles, further membrane heterogeneity is created by generation of PI(3,4)P_2_ around the forming clathrin-coated vesicle (Figure 1D) (Posor et al., 2013). PIK3C2α is recruited to the clathrin lattice *via* interactions with clathrin and PI(4,5)P_2_, where it metabolizes PI(4,5)P_2_ to PI(3,4)P_2_ in conjunction with the 5-phosphatases ORCL and synaptojanin-1 (Posor et al., 2013; He et al., 2017; Schöneberg et al., 2017). This distinct phospholipid environment promotes the recruitment and activation of the BAR-domain containing protein SNX9 that interacts with actin-branching activators and dynamin (Figure 1D) (Posor et al., 2013; Lo et al., 2017; Schöneberg et al., 2017) and provides constricting force at the neck of the endocytic vesicle (Shin et al., 2008; Ferguson et al., 2009).

The final alteration in membrane heterogeneity in clathrin-dependent endocytosis serves to remove clathrin and adaptor proteins from the endocytic vesicle, ensuring scission from the plasma membrane and entry into the endocytic trafficking pathway. Endophilin is a further BAR-domain containing protein that is recruited to dynamin at the neck of the endocytic vesicle (Perera et al., 2006; Ferguson et al., 2009). Endophilin, as with SNX9 and other BAR-domain containing proteins, can deform membranes to assist dynamin in vesicle scission (Figure 1E) (Itoh et al., 2005). Endophilin also directly modifies the membrane composition of the endocytic vesicle by recruiting the 5-phosphatase synaptojanin-1 to the neck region of the vesicle immediately prior to fission. Synaptojanin-1 metabolizes PI(4,5)P_2_ to PI(4)P, which makes further clathrin and AP-2 binding unfavorable (Perera et al., 2006; Milosevic et al., 2011). Auxilin, which is recruited to the mature pit by dynamin and binding to enriched PI(4)P, recruits the uncoating chaperone HSC70, ultimately leading to uncoating of the clathrin-adaptor complex from the endocytic vesicle and release of the vesicle into the cell (Massol et al., 2006; Guan et al., 2010). Recruitment of the 5-phosphatase ORCL to the neck of the clathrin-coated pit by SNX9 immediately prior to fission further stimulates vesicle uncoating (Nández et al., 2014).

Key points:

• Clathrin-dependent endocytosis is regulated by membrane heterogeneity at every step.
• PI(4,5)P_2_ enrichment at the plasma membrane generates membrane curvature and a distinct domain within the plasma membrane, which eventually recruits clathrin.
• PI(4,5)P_2_ enrichment recruits AP-2 and phosphatidylinositol kinase type-I, further promoting high local PI(4,5)P_2_ concentration.
• PI(4,5)P_2_ and clathrin recruit PIK3C2α, which produces PI(3,4)P_2_, resulting in PI(3,4)P_2_ enrichment around the clathrin-coated region while leaving PI(4,5)P present in the neck region.
• PI(4,5)P_2_ in the neck region recruits dynamin, SNX9, endophilin and branched actin, ultimately leading to vesicle scission and uncoating of clathrin from the vesicle.

### Clathrin-Independent Endocytosis

Clathrin-independent endocytosis represents multiple endocytic pathways with the common factor being that they do not rely on clathrin. The major difference in the initiation of endocytosis between clathrin-mediated and clathrin-independent endocytosis is the local microenvironment at the site of internalization, making membrane heterogeneity a key determinant of the endocytic mode responsible for internalizing cargo.

### Fast Endophilin-Mediated Endocytosis

**FIGURE 2:**
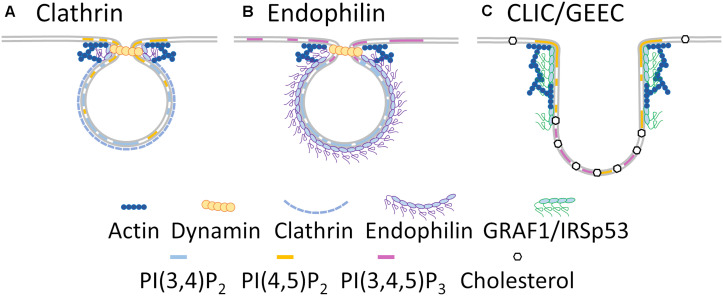
Membrane heterogeneity regulates endocytic specificity. Clathrin dependent endocytosis **(A)** occurs in PI(3,4)P_2_ and PI(4,5)P_2_ enriched regions of the plasma membrane and requires endophilin, dynamin and actin recruitment for endosomal scission. Fast endophilin-mediated endocytosis **(B)** occurs in PI(3,4)P_2_ and PI(3,4,5)P_3_ enriched regions of polarized membranes, with endophilin likely forming the coat around the endosome, and branched actin and dynamin required for endosomal scission. CLIC/GEEC endocytosis **(C)** occurs in cholesterol, PI(4,5)P_2_ and PI(3,4,5)P_3_ enriched regions of the plasma membrane and relies on branched actin and GRAF1/IRSp53 BAR -domain containing proteins for endosomal scission.

Endophilin plays an important role in vesicle scission and uncoating and is a central regulator of the fast endophilin-mediated endocytosis pathway (Boucrot et al., 2015). While clathrin-dependent endocytosis occurs across the entire plasma membrane at regions of PI(4,5)P_2_ enrichment (Figure 2A), fast endophilin-mediated endocytosis occurs within PI(3,4,5)P_3_-rich sites in response to activation of receptors (Figure 2B) (Leonard et al., 2008; Boucrot et al., 2015).

CDC42 activity initiates fast endophilin-mediated endocytosis through the recruitment of the BAR-domain containing proteins FBP17 and CIP4, which in turn recruit the 5-phosphatase SHIP2 (Chan Wah Hak et al., 2018). Activated SHIP2 metabolizes PI(3,4,5)P_3_ to PI(3,4)P_2_ at the cell leading edge (Pesesse et al., 2001) resulting in the recruitment of lamellipodin and eventually endophilin (Boucrot et al., 2015; Chan Wah Hak et al., 2018). Cycling of CDC42 activation leads to constant assembly/disassembly of membrane patches primed for endophilin recruitment. These are only stabilized if ligand-bound receptors are present, in which case the endophilin-enriched membrane patch proceeds through endocytosis (Chan Wah Hak et al., 2018). Interestingly, in clathrin-dependent endocytosis, endophilin localizes to the highly curved, PI(4,5)P_2_ enriched neck region of the endocytic vesicle (Perera et al., 2006; Milosevic et al., 2011). During fast endophilin-mediated endocytosis, endophilin is concentrated in a completely different PI(3,4)P_2_-enriched membrane environment at the leading edge of the cell (Boucrot et al., 2015). This highlights how membrane heterogeneity ultimately controls how one protein can regulate two endocytic modes (Figures 2A,B).

In contrast to clathrin-dependent endocytosis where the membrane is held in a defined, rigid conformation by the clathrin coat, endophilin-mediated endocytosis requires a malleable membrane for formation and scission of the vesicle from the plasma membrane. Twenty BAR-domain containing proteins have been identified in endophilin-enriched membrane patches thus far, strongly indicating that local membrane bending and remodeling is the critical factor in forming endosomes in fast endophilin-mediated endocytosis (Chan Wah Hak et al., 2018).

Key points:

• Fast endophilin-mediated endocytosis is initiated within PI(3,4,5)P_3_ areas of the cell.
• CDC42 assembles primed membrane patches for fast endophilin-mediated endocytosis, recruiting BAR-domain proteins and the 5-phosphatase SHIP2.
• If ligand: receptor binding occurs, SHIP2 is activated leading to rapid metabolism of PI(3,4,5)P_3_ to PI(3,4)P_2_.
• PI(3,4)P_2_ enriched patches recruit lamellipodin and endophilin, leading to cargo endocytosis.

### CLIC/GEEC Endocytosis

CLIC/GEEC endocytosis is predominantly defined as being a clathrin-independent, dynamin-independent endocytic pathway (Doherty and Lundmark, 2009). Similar to fast endophilin-mediated endocytosis, CLIC/GEEC endocytosis occurs at the leading edge or polarized membranes of the cell in a CDC42 and BAR-domain containing protein dependent manner, and is notable for the formation of pronounced membrane tubules carrying cargo (Howes et al., 2010; Francis et al., 2015; Rossatti et al., 2019). While clathrin-dependent and fast endophilin-mediated endocytosis predominantly regulate receptor internalization, CLIC/GEEC endocytosis regulates receptor internalization (Kumari and Mayor, 2008; Rossatti et al., 2019), toxin uptake (Lundmark et al., 2008; Romer et al., 2010) and fluid-phase endocytosis (Howes et al., 2010).

CLIC/GEEC endocytosis occurs in either PI(4,5)P_2_ or PI(3,4,5)P_3_ enriched membranes and relies more on plasma membrane cholesterol than clathrin dependent or fast endophilin mediated endocytosis (Figure 2C). The presence of cholesterol within the plasma membrane is also critical for CDC42 activation and subsequent actin polymerisation. Consequently, mild cholesterol depletion abolishes CLIC/GEEC endocytosis while leaving clathrin-dependent endocytosis unperturbed (Chadda et al., 2007). Active CDC42 recruits the BAR-domain containing proteins GRAF-1 and/or IRSp53 and the actin nucleation complex Arp2/3 to the plasma membrane which together induce membrane curvature and tubulation that is characteristic of the CLIC/GEEC pathway (Francis et al., 2015; Sathe et al., 2018). GRAF-1 binds PI(4,5)P_2_ (Lundmark et al., 2008) while IRSp53 binds PI(3,4,5)P_3_ and PI(4,5)P_2_ (Suetsugu et al., 2006), indicating that CLIC/GEEC endocytosis may occur at wider range of membrane environments than fast endophilin-mediated endocytosis, perhaps to account for its role in large-scale membrane turnover (Howes et al., 2010; Francis et al., 2015). CLIC/GEEC endocytosis is also sensitive to local membrane tension, its rate increasing with decreasing membrane tension, *via* the mechanotransducer vinculin (Thottacherry et al., 2018). Phosphatidylserine clustering in the inner leaflet of the plasma membrane is capable of promoting membrane curvature and endocytosis (Hirama et al., 2017). Conversely, cholesterol limits phosphatidylserine clustering in the plasma membrane, preventing spontaneous endocytosis (Hirama et al., 2017; Hirama and Fairn, 2018). However, molecular dynamics simulations indicate cholesterol itself induces membrane curvature and associates with phosphatidylserine in highly curved regions in asymmetric membranes (Yesylevskyy et al., 2013), indicating a complex relationship between cholesterol and phosphatidylserine in endocytosis. We therefore speculate CLIC/GEEC endocytosis may rely on the functional interrelation of cholesterol and phosphatidylserine at the plasma membrane and the interplay of both to induce the local membrane curvature required for endosome formation.

A critical difference between fast endophilin-mediated endocytosis and CLIC/GEEC endocytosis is the lack of dynamin-dependence for vesicle scission off the plasma membrane in the latter. A confluence of BAR-domain containing proteins deforming CLIC/GEEC membrane tubules coupled with tubule constriction by branched actin fibers may provide the physical force needed for endosome scission in the absence of dynamin (Sathe et al., 2018). Cholesterol, in coordination with actin polymerisation, greatly enhances scission of membrane tubules formed during CLIC/GEEC endocytosis (Romer et al., 2010), and is required for CDC42 activation dependent actin polymerisation on PI(4,5)P_2_-enriched membranes (Chadda et al., 2007). It is likely that cholesterol and PI(4,5)P_2_ in CLIC/GEEC membrane tubules is an absolute requirement for the recruitment of BAR-domain containing proteins required for scission to release endosomes. Specific membrane heterogeneity – in terms of membrane composition, curvature and tension – is therefore required for not only initiating CLIC/GEEC endocytosis, but also in ensuring scission of CLIC/GEEC endocytic tubules.

Key points:

• CLIC/GEEC endocytosis occurs in cholesterol, PI(3,4,5)P_3_ or PI(4,5)P_2_ enriched regions of the cell leading edge or polarized membranes of the cell.
• Cholesterol, in conjunction with phosphatidylserine, may induce the membrane curvature required for initiation of CLIC/GEEC endocytosis.
• CDC42 recruits GRAF1 and IRSp53, which in conjunction with branched actin induce membrane tubulation.
• GRAF1 and IRSp53 can bind PI(4,5)P_2_ and/or PI(3,4,5)P_3_, leading to induction of CLIC/GEEC endocytosis in a wide range of membrane patches on the cell leading edge.
• Cholesterol and branched actin are capable of inducing membrane tubule scission, leading to the formation of endocytic carriers in CLIC/GEEC endocytosis.

## Endosomal Sorting

**FIGURE 3:**
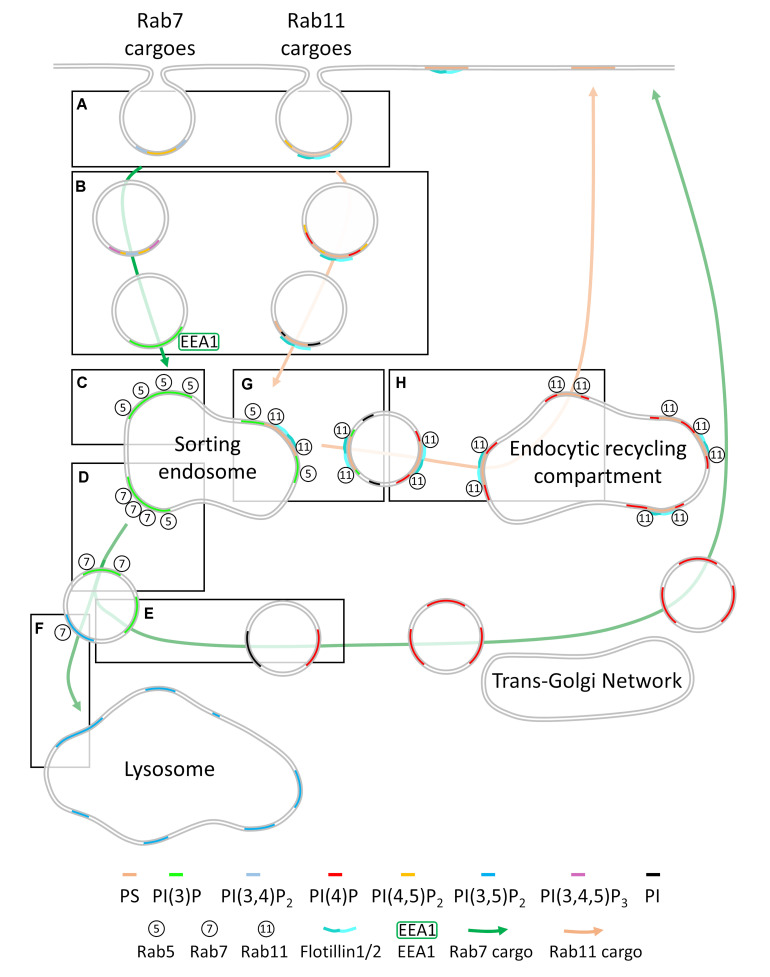
Membrane heterogeneity regulates cargo sorting throughout the endocytic network. **(A)** Cargoes are segregated into endocytic pits containing a specific combination of phosphoinositides (PI, left) or phosphoinositides and phosphatidylserine (PS, right) at the plasma membrane. This ensures cargoes are kept segregated after having been internalized. Endosomes containing only phosphoinositides become enriched in PI(3)P at this point (left) while phosphoinositides on phosphatidylserine containing endosomes are de-phosphorylated **(B)**. Cargo destined for Rab7-dependent trafficking enters the Rab5^+^ sorting endosome, where it resides in a PI(3)P-enriched membrane domain **(C)**. This PI(3)P enrichment recruits Rab7 and leads to the inactivation of Rab5, maturing into a Rab7^+^ late endosome **(D)**. PI(4,5)P_2_ is generated on the Rab7^+^ late endosome, allowing recruitment of retromer/retriever/CCC factors that retrieve the cargo, metabolize the PI(4,5)P_2_ and PI(3)P to phosphoinositide and PI(4)P, displacing Rab7 and allowing cargo trafficking to the *trans*-Golgi network, where it can be re-secreted to the plasma membrane **(E)**. Rab7-dependent cargoes that are not trafficked to the *trans*-Golgi network are trafficked to the lysosome for degradation **(F)**. Cargoes entering the cell via the phosphatidylserine-enriched endocytic pathway fuse with the Rab5^+^ sorting endosome, where their membrane domains are enriched in PI(3)P, leading to recruitment of Rab11 and scission from the Rab5^+^ sorting endosome **(G)**. Following scission, the PI(3)P on the Rab11^+^ vesicle is metabolized to PI(4)P, allowing fusion with the Rab11^+^ endocytic recycling compartment and re-delivery to the plasma membrane **(H)**.

Endocytosed cargoes are sorted via three main routes that result in different fates: (1) recycling to the plasma membrane via retromer/retriever and the *trans*-Golgi network, (2) degradation by the lysosome, or (3) recycling to the plasma membrane *via* Rab11 dependent recycling endosomal pathway (Figure 3). Rab7 regulates formation of the late endosome, from which retromer, retriever and COMMD/CCDC22/CCDC93 (CCC)-dependent cargo retrieval and recycling occurs (McNally et al., 2017; Purushothaman et al., 2017; Singla et al., 2019) or from which cargoes are directed to the lysosome for degradation (Guerra and Bucci, 2016). Rab4 is predominantly localized to the Rab5^+^ sorting endosomal compartment (Sönnichsen et al., 2000) and can regulate cargo transfer into both Rab11 recycling and cellular degradative pathways (McCaffrey et al., 2001; Nag et al., 2018). Little is known about the composition of membranes that recruit Rab4, however. Rab11 regulates trafficking of cargoes to the endocytic recycling compartment for direct recycling to the plasma membrane (Takahashi et al., 2012). Entry into these pathways is regulated by the Rab5^+^ sorting endosome, which has been historically viewed as where the sorting decisions for the cell are carried out (Jovic et al., 2010; Naslavsky and Caplan, 2018).

Membrane heterogeneity acts as a decisive regulator of each step of the sorting process, from the plasma membrane and throughout the endosomal network. Recent evidence indicates that cargoes are pre-sorted into distinct plasma membrane environments and that the sorting fate of a protein is either determined with the formation of the endocytic coat or immediately following endocytosis (Sigismund et al., 2005, 2008; Lakadamyali et al., 2006; Qian et al., 2007; Leonard et al., 2008; Leren, 2014; Sposini et al., 2017; Redpath et al., 2019). Membrane heterogeneity plays a decisive role in sorting cargoes toward the Rab7 or Rab11 pathways. As cargoes remain segregated following endocytosis (Lakadamyali et al., 2006; Leonard et al., 2008; Franke et al., 2019), we speculate that the membrane lipids of each type of endocytic vesicle could also remain segregated following fusion with the Rab5^+^ endosome. From here, these distinct membrane domains undergo specific changes in their composition that lead to recruitment of either Rab7 or Rab11. Rab7 leads to recruitment of retromer/retriever complexes for retrograde recycling or of the endosomal sorting complex required for transport (ESCRT) for lysosomal degradation. Recruitment of Rab11 leads to Rab11-dependent recycling (Fairn et al., 2011; Franco et al., 2014; Takatori et al., 2016; Campa et al., 2018; Cullen and Steinberg, 2018; Redpath et al., 2019). SNAREs, the protein complexes responsible for fusing cellular membranes together also reside in specific regions of heterogenous membranes. The role of SNAREs in targeting and fusion of subcellular membranes extend beyond the scope of this review but is extensively is reviewed in Jahn and Scheller (2006) and Dingjan et al. (2018).

### Sorting for Retromer/Retriever/CCC-Dependent Recycling or Lysosomal Degradation

Even though the fate of cargoes is presumably determined prior to or during endocytosis, trafficking is required to be highly modular in order to adapt to cellular conditions. As such, trafficking via the Rab5 to Rab7 endocytic pathway can result in either recycling *via* the retromer/retriever pathway or degradation in the lysosome (Figures 3A–F) (Guerra and Bucci, 2016). Membrane domains with distinct phosphoinositide contents control entry into and sorting along the Rab5 to Rab7 pathway. Cargoes targeted to this route enter the Rab5^+^ sorting endosome in PI3P enriched domains distinct from those of Rab11-dependent recycling cargoes, which are enriched in phosphatidylserine (Figures 3A,B – left) (Lakadamyali et al., 2006; Leonard et al., 2008; Tanaka et al., 2016). These cargoes remain in the Rab5^+^ sorting endosomes, which mature into Rab7^+^ late endosomes. They are then clustered in ESCRT-rich domains within Rab7^+^ membranes and transported into lysosomes in which they are degraded (Rink et al., 2005). However, depending on the cellular conditions, cargoes typically targeted to the degradative pathway can be redirected for recycling *via* the *trans*-Golgi by the recruitment of the retriever and CCC complexes within subdomains of the Rab7^+^ endosome (Burd and Cullen, 2014; McNally et al., 2017; Singla et al., 2019).

Following internalization, cargoes addressed to Rab7^+^ endosomes are segregated from those cargoes destined for Rab11-depenent recycling and rapidly enter EEA1^+^ compartments on their way to the Rab5^+^ sorting endosome (Lakadamyali et al., 2006). This step relies on membrane heterogeneity, as residual phosphoinositides from the endocytic pit are metabolized to PI(3)P (Christoforidis et al., 1999). PI(4,5)P_2_ on the endosome (either immediately, prior to, or following fission from the plasma membrane) is phosphorylated to PI(3,4,5)P_3_ by PI3Kβ (Shin et al., 2005). PI(3,4,5)P_3_ is subsequently metabolized to PI(3,4)P_2_ and PI(3)P by OCRL and INPP4A/B (Shin et al., 2005; Vicinanza et al., 2011). Residual PI(3,4)P_2_ from the endocytic pit is similarly metabolized to PI(3)P (Shin et al., 2005, Figures 3A,B – left).

These PI(3)P-positive membranes promote the recruitment of EEA1 which triggers cargo entry into the Rab5^+^ sorting endosome (Christoforidis et al., 1999; Murray et al., 2016). EEA1 acts as a fusogenic tether (Christoforidis et al., 1999; Murray et al., 2016) and simultaneously binds both PI(3)P and Rab5 to bring PI(3)P^+^ endosomes together with Rab5^+^ sorting endosomes (Figures 3B,C – left) (Lawe et al., 2002; Murray et al., 2016). EEA1 binding to the membrane of endocytic vesicles after internalization represents a crucial difference in the pre-Rab5^+^ sorting of Rab11- and Rab7-dependent cargoes. Typically, while epidermal growth factor (EGF) remains associated with EEA1^+^ vesicles, transferrin either only spuriously interacts with or is rapidly sorted through this population of endocytic vesicles (Leonard et al., 2008). It follows that EEA1 knockdown reduces degradation of EGF-stimulated EGF receptor, while transferrin uptake and recycling is unaffected (Leonard et al., 2008; Navaroli et al., 2012) (Figure 3B). Interestingly, our own studies of TCR, another Rab11-dependent recycling cargo, reveals similar short interactions with Rab5^+^ early endosomes following endocytosis (Redpath et al., 2019), indicating that recycling cargoes may only very transiently interact with early endosomal compartments.

Similarly to EEA1, sorting nexin 15 (SNX15) binds to and promotes the formation of heterogeneous membrane domains to ensure correct sorting of cargoes trafficking toward the lysosome immediately following clathrin-dependent endocytosis (Danson et al., 2013; Flores-Rodriguez et al., 2015). SNX15 can drive entry of EGF into EEA1^+^ and Rab5^+^ endosomes through the simultaneous ability to interact with clathrin and PI(3)P (Danson et al., 2013; Flores-Rodriguez et al., 2015; Chandra et al., 2019). SNX15 also interacts with the ESCRT protein family, which deform membranes for formation of multi-vesicular bodies (a prelude to lysosomal trafficking) (Flores-Rodriguez et al., 2015).

Rab5^+^ sorting endosomes mature into late endosomes over time by acquiring Rab7 and losing Rab5 (Figures 3C,D). This transition relies on membrane heterogeneity, and more specifically on timed and localized enrichment in PI(3)P. Regions of Rab5^+^ sorting endosome get progressively enriched in PI(3)P, leading to recruitment of Rab7 and inactivation of Rab5 (Figure 3D) (Rink et al., 2005). These specific membranes within the Rab5^+^ endosome have a high PI(3)P content (Figure 3C) (Gillooly et al., 2003), either because they result from fusion with EEA1 endosomes or because of Rab5-dependent recruitment of the phosphoinositide-3 kinase VPS34, which generates PI(3)P from PI (Shin et al., 2005). Enlargement of Rab5 endosomes – likely due to continued delivery of cargo-containing endocytic vesicles to the Rab5^+^ compartment – leads to recruitment of the protein SAND-1/Mon1, which subsequently displaces Rab5 activating factors and recruits Rab7 to the endosome (Poteryaev et al., 2010). High levels of PI(3)P also allow the binding of TBC-2, which is an inhibitor of Rab5 (Law et al., 2017). Once recruited on Rab5^+^ endosomes, Rab7 acts to negatively regulate further PI(3)P synthesis by recruiting WDR91 (Liu et al., 2016, 2017). Sufficient PI(3)P must, however, remain on the endosome to recruit the Rab7 inactivating factor, Armus for later termination of Rab7 activity (Jaber et al., 2016). Too little PI(3)P generation leads to Rab5 and Rab7 hyperactivation and ultimately defective cargo trafficking to the lysosome (Jaber et al., 2016). Conversely, cargoes fail to leave early endosomes in presence of too high levels of PI(3)P (Liu et al., 2017).

PI(3)P enriched microdomains in the Rab7^+^ late endosomal membrane are also central to recovering cargoes and circumventing lysosomal degradation for re-delivery to the plasma membrane, directly or through the *trans* Golgi network (Figure 3D). Three protein complexes have been identified to stimulate this recovery *via* recognition of specific sequences on the cargo: retromer, retriever and the CCC complex (McNally et al., 2017; Purushothaman et al., 2017; Singla et al., 2019). Retromer and retriever are formed by specific sorting nexin family members and three vacuolar protein sorting (VPS) family subunits. Both retromer and retriever bind the CCC protein complex, which directly regulates endosomal PI(3)P levels (Singla et al., 2019). Retromer is recruited to the Rab7 endosomal membrane *via* direct interaction between Rab7 and the retromer VPS subunits, and PI(3)P binding by the SNX subunits (Rojas et al., 2008). Neither the VPS or SNX subunits of retriever associate with Rab7 or PI(3)P, respectively, despite residing on the same endosomal membrane domains as retromer (McNally et al., 2017). How precisely retriever is targeted to the same membrane domain as retromer is not yet fully understood, however, retriever associates with the CCC complex which itself has PI(3)P binding capacity (McNally et al., 2017). Alongside PI(3)P, PI(4,5)P_2_ plays an important role in the recycling of cargos through the retromer/retriever pathway. PI(4,5)P_2_ must be generated on the Rab7^+^ endosomal membrane by the phophoinositide-5 kinase PIPKIγi5 for cargo entry and direct retromer recruitment (Sun et al., 2020). Furthermore, binding of the COMMD1 subunit of the CCC complex to PI(4,5)P_2_ is required for cargo recovery to the *trans*-Golgi network (Stewart et al., 2019), providing a potential link between retriever and the PI(3)P/PI(4,5)P_2_-enriched membrane domain utilized by retromer for cargo recovery (Figures 3D,E – left).

Multiple local phosphoinositide changes in the membrane domains defined by retromer/retriever are required to facilitate cargo transport toward the *trans*-Golgi network (Figure 3E – right). Prior to scission from the Rab7^+^ late endosome, the actin-branching activating complex WASH localizes to the PI(3)P-enriched retrieval subdomains (Gomez and Billadeau, 2009; Cullen and Steinberg, 2018; Singla et al., 2019). Actin polymerization downstream of WASH leads to scission of the retromer membrane domain from the Rab7^+^ late endosome (Gomez and Billadeau, 2009). After scission, PI(3)P is metabolized to PI by the 3-phosphatase MTMR2, which leads to a reduction in WASH activation and displacement of actin from the retromer/retriever membrane domain (Cao et al., 2007; Singla et al., 2019). Without PI(3)P reduction, actin accumulates on endosomal membranes and cargo degradation is increased while recycling decreases (Bartuzi et al., 2016; Singla et al., 2019). Local membrane composition further contributes to regulate the transport of retromer cargoes toward the *trans*-Golgi network. Following scission from the late endosome, the subunit SNX6 links the retromer membrane domain with the molecular motor dynein, resulting in cargo transport (Hong et al., 2009; Wassmer et al., 2009; Purushothaman et al., 2017). SNX6 then binds enriched PI(4)P membranes in the *trans*-Golgi network, which subsequently causes release of the molecular motor dynein and cargo delivery into the *trans*-Golgi network, from where re-secretion can finally occur (Niu et al., 2013).

Cargoes not recovered from the late endosome by retromer, retriever or the CCC complex are clustered in membrane domains enriched in the ESCRT complex, which initiates their transport to the lysosome for degradation (Figure 3F) (Rojas et al., 2008; Bartuzi et al., 2016; McNally et al., 2017; Cullen and Steinberg, 2018). PI(3,5)P_2_ is generated from residual PI(3)P by PIK*fyve*, which is critical for maintaining the membrane integrity of late Rab7^+^ endosomes and lysosomes (Ikonomov et al., 2002, 2006; Bissig et al., 2017). Rab7^+^ lysosomes represent terminal compartments in which cargoes not destined for recycling are degraded (Humphries et al., 2011). The interaction between Rab7 and PI(3,5)P_2_, or how PI(3,5)P_2_ recruits other requisite lysosomal proteins is not yet known.

Key points:

• The Rab5/Rab7 endocytic pathway relies on tightly controlled PI(3)P enrichment.
• PI(3)P recruits Rab7 to the Rab5^+^ sorting endosome and recruits Rab5 inactivating factors.
• PI(3)P recruits retromer/retriever/CCC subunits required to facilitate cargo recovery to the *trans*-Golgi network.
• PI(3)P recruits actin branching factors to facilitate endosomal scission from the late endosome.
• PI(4,5)P is enriched in retromer/CCC-bound membrane domains and is required for cargo recovery.
• Once vesicles targeting the Golgi have left the Rab7^+^ endosome, PI(3)P is metabolized to PI.
• PI(4)P from the *trans*-Golgi network recruits retromer subunits, resulting in cargo delivery.
• PI(3)P is converted to PI(3,5)P_2_ during lysosomal maturation, resulting in cargo degradation.

### Sorting for Rab11-Dependent Recycling

Rab11-dependent recycling relies on delivery of endocytic cargoes to the Rab11^+^ peri-nuclear endocytic recycling compartment, from where they are re-delivered to the plasma membrane (Ullrich et al., 1996; Redpath et al., 2019). The exact mechanism of cargo sorting before and into the Rab11^+^ recycling endosome is not clearly established or understood in the literature compared to the relatively well-described retromer/retriever-dependent recycling pathway. However, recent studies have begun to reveal a clearer picture. Cargoes destined for Rab11^+^-dependent recycling can be internalized *via* clathrin-dependent or independent pathways (Ullrich et al., 1996; Compeer et al., 2018). Transferrin and EGF, both internalized via clathrin-dependent endocytosis, are found segregated in the plasma membrane then trafficked via a Rab11 or Rab7-dependent pathways, respectively. This delineation at level of the plasma membrane is clearly of functional importance highlighted by the subsequent divergent sorting into the Rab11 or Rab7 pathways (Figure 3A – right) (Lakadamyali et al., 2006; Leonard et al., 2008). Immediately following endocytosis, cargoes endocytosed by clathrin-dependent or independent modes and destined for Rab11-dependent recycling appear to be committed to this fate (Lakadamyali et al., 2006; Leonard et al., 2008; Redpath et al., 2019), supporting the notion that a distinct plasma membrane environment must exist to facilitate sorting of Rab11-dependent recycling cargo early on.

Membrane heterogeneity is modulated *via* phosphoinositide switching on phosphatidylserine-enriched membranes throughout the endocytic pathway and is therefore critical for cargo progression throughout the Rab11-dependent recycling pathway (Figures 3A,B,G,H – right). The exact properties of the plasma membrane domains where Rab11-dependent recycling cargoes reside is not yet clearly defined. There is, however, substantial evidence to formulate a speculative model that is centered around phosphatidylserine-enriched membranes. Rab11-family interacting proteins (Rab11-FIPs) bind distinct phosphatidylserine membranes (Baetz and Goldenring, 2014) and link Rab11 to EHDs, dynein, SNAREs, VPS and SNX proteins to facilitate cargo exit from sorting endosomes and re-delivery to the plasma membrane (Naslavsky et al., 2006; Horgan et al., 2010; Solinger et al., 2020). This indicates that phosphatidylserine-enriched membranes are critical for assembling the protein machinery required for Rab11-dependent recycling. Phosphatidylserine attracts specific cargo binding and is present on the cytosolic leaflet of the plasma membrane and throughout the Rab5 to Rab11 endocytic pathway (Figure 3A – right) (Yeung et al., 2008; Hirama et al., 2017). Phosphatidylserine is capable of inducing endocytosis without canonical clathrin-dependent or clathrin-independent endocytic regulators, indicating that phosphatidylserine enriched microdomains in the plasma membrane are themselves primed for endocytosis (Hirama et al., 2017). Phosphatidylserine is maintained strictly on the cytoplasmic face of endosomes throughout the entire Rab11^+^ endocytic recycling pathway, and this enrichment is an absolute requirement for cargo recycling to occur (Figures 3A,B,G,H) (Chen et al., 2010; Fairn et al., 2011; Uchida et al., 2011; Lee et al., 2015; Tanaka et al., 2016). Transferrin is processed through the Rab11-dependent recycling route. It co-localizes with phosphatidylserine from the earliest stages of endocytosis, in contrast to EGF which traffics via the Rab5 to Rab7 degradative/recycling pathway and is found only transiently in a subset of phosphatidylserine enriched endosomes (Figure 3A – right) (Tanaka et al., 2016). Cargo persistence in phosphatidylserine enriched endosomes could be required from the initiation of endocytosis to ensure sorting into Rab11-positive recycling endosomes. This illustrates that segregation of cargo into phosphatidylserine-enriched membrane domains at the level of the plasma membrane facilitates later sorting into the Rab11-dependent recycling pathway.

Immediately following endocytosis, membrane heterogeneity continues to play a role in determining sorting of cargoes for Rab11-dependent recycling. Tightly regulated dephosphorylation of phosphoinositides in time and space is a critical requirement for Rab11-dependent recycling cargoes. Following clathrin-dependent or -independent endocytosis, the 5-phosphatase ORCL and 4-phosphatase Sac2 are recruited to newly formed endosomes, which sequentially metabolize residual PI(4,5)P_2_ from endocytic pit formation to PI(4)P and then PI (Figure 3B – right) (Nakatsu et al., 2015). Generation of PI(4)P by Sac2 is required for subsequent sorting of transferrin out of the Rab5^+^ endosome. By contrast, EGF does not require Sac2 to exit Rab5^+^ sorting endosomes (Hsu et al., 2015), indicating that heterogeneous generation of PI within the Rab5^+^ sorting endosome is crucial for cargo discrimination. It has not yet been determined if residual PI(3,4)P_2_ from the endocytic pit is metabolized to PI(3)P or PI in endosomes destined for Rab11 recycling.

Cargoes that are sorted from Rab5 to Rab7 stably associate with EEA1^+^ and Rab5^+^ endosomes rapidly following endocytosis, while the EEA1^+^ intermediate is dispensable for transferrin sorting (Leonard et al., 2008; Navaroli et al., 2012). Compared to the Rab5-to-Rab7 cargoes, cargo destined for Rab11-dependent recycling associates only transiently with the Rab5^+^ sorting endosome (Lakadamyali et al., 2006; Leonard et al., 2008; Redpath et al., 2019). Strikingly, despite this transient association, cargoes eventually sorted to both Rab11^+^ and Rab7^+^ compartments can be present within the same Rab5^+^ endosome (Figures 3D,G) (Franke et al., 2019), highlighting incredible heterogeneity in membrane domains within a relatively small intracellular organelle. In contrast to the Rab5 to Rab7 sorting domain, in which PI(3)P generated by VPS34 biogenesis of the Rab5^+^ endosome facilitates recruitment of Rab7 (Shin et al., 2005), inactive Rab11 is already present on peripheral Rab5^+^ endosomes and is activated by local generation and enrichment of PI(3)P by PI3K-C2α (Figure 3G – left) (Franco et al., 2014; Campa et al., 2018). Once activated by local PI(3)P enrichment, recruitment of the SNAREs, EHD1, sorting nexins and VPS proteins of the FERARI complex to Rab11 facilitates removal of cargoes from the Rab5^+^ endosome for subsequent recycling (Figures 3G,H) (Campa et al., 2018; Solinger et al., 2020).

There is strong evidence highlighting the need for membrane heterogeneity within the Rab11^+^ recycling network for cargo progression along the endocytic recycling axis. Activation of Rab11 recruits the phosphatidylinositol 3-phosphatase MTM1 to PI(3)P enriched domains, where it converts PI(3)P to unphosphorylated phosphoinositide (PI), which leads to the rapid dissociation of Rab11^+^ vesicles from the Rab5^+^ endosome (Figure 3G – right) (Ketel et al., 2016; Campa et al., 2018). PI(3)P enrichment is critical to vesiculation and removal of the Rab11^+^ domain from the Rab5^+^ compartment (Campa et al., 2018). PI(3)P further promotes the association of Rab5^+^ endosomes with fission factors and the microtubule motor dynein – through interactions with the BAR-domain containing protein SNX4 and/or SNX1 and Rebenosyn-5. Rab-FIP5 is central for the interaction of Rab11 with these fission factors, indicating phosphatidylserine enrichment is still required within endosomal membrane to facilitate the Rab5-to-Rab11 sorting events (Baetz and Goldenring, 2014; Campa et al., 2018; Solinger et al., 2020). Dynein provides the force for vesicle fission and delivers the newly formed vesicle to the endocytic recycling compartment (Traer et al., 2007; Horgan et al., 2010). The newly formed vesicular PI is then phosphorylated to PI(4)P by PI4Kα, which leads to delivery of the vesicle to the perinuclear endocytic recycling compartment and to the recruitment of the exocyst machinery required for cargo re-delivery to the plasma membrane (Figure 3H) (Ketel et al., 2016). Interestingly, recycling cargoes internalized by both clathrin-dependent and -independent modes ultimately traffic to the endocytic recycling compartment, yet remain segregated within this compartment (Xie et al., 2016), indicating that within the Rab11^+^ recycling network heterogeneity membrane domains may be required for cargo segregation.

Amongst the rapidly changing and tightly regulated environment of membrane phosphoinositides on Rab11^+^ endosomes, phosphatidylserine remains enriched and precisely oriented on the cytoplasmic face of recycling cargo membrane domains. Phosphatidylserine is present in the Rab5^+^ sorting endosome, Rab11^+^ vesicles and the Rab11^+^ endocytic recycling compartment (Figure 3) (Chen et al., 2010; Baetz and Goldenring, 2014; Lee et al., 2015). Disruption of multiple confirmed or suspected phosphatidylserine flippases or translocases abolishes recycling and leads to accumulation of cargoes in EEA1/Rab5^+^ early/sorting endosomes, in Rab11^+^ vesicles and in endocytic recycling compartments (Chen et al., 2010; Lee et al., 2015; Tanaka et al., 2016). Furthermore, cytoplasmic orientation of phosphatidylserine is required for recruitment of the membrane deforming protein EHD1 to the endocytic recycling compartment, where it induces membrane tubulation to allow cargo exit for recycling (Lee et al., 2015; Deo et al., 2018).

Rab11-dependent recycling membrane domains are enriched in the membrane domain organizing proteins: flotillin-1 and flotillin-2 (flotillins hereafter), which regulate the recycling of a wide range of cargoes (Solis et al., 2013; Hülsbusch et al., 2015; Bodrikov et al., 2017; Compeer et al., 2018). Flotillins define cholesterol-rich membrane domains throughout the plasma membrane and endocytic recycling network and require the presence of phosphatidylserine for localization to these domains (Maekawa and Fairn, 2015). Flotillins interact with SNX4 and Rab11 along membrane tubules and within the endocytic recycling compartment, and are required for the sorting of transferrin and the T-cell receptor into Rab11^+^ endosomes (Solis et al., 2013; Redpath et al., 2019). New data from our group has demonstrated that flotillins interact extremely rapidly with Rab5^+^ endosomes and then accumulate in Rab11^+^ endosomes (Redpath et al., 2019). Taken together, we have proposed that flotillins-based domains act as entry doors into the Rab5^+^-Rab11^+^ recycling endosomal network. These domains are maintained on Rab5^+^ endosomes and are unique from the surrounding Rab5^+^ endosomal membrane. Eventually, they could then facilitate interaction with the FERARI complex of sorting nexins, rabenosyn-5, EHD1, SNAREs, and VPS proteins, dynein and Rab11 to ensure efficient cargo sorting to the recycling endosomal compartment (Figures 3B,G,H).

Sorting for Rab11-dependent recycling is clearly critically dependent on membrane heterogeneity. Phosphoinositides are rapidly catabolised, enriched and converted to ensure recruitment of the requisite sorting factors at the correct time; phosphatidylserine is maintained in a strict cytoplasmic orientation throughout this rapidly changing phosphoinositide environment to regulate cargo endocytosis, delivery to and recycling from the Rab11^+^ endocytic recycling compartment. Finally, membrane-organizing proteins may act to maintain microdomains throughout the Rab11^+^ recycling network to ensure cargo is efficiently sorted throughout this entire process.

Key points:

• Phosphatidylserine is enriched throughout the Rab11^+^ endocytic recycling pathway.
• Residual plasma membrane PI(4,5)P is metabolized to PI prior to Rab11 recycling cargo entry into the Rab5^+^ sorting endosome.
• A localized enrichment of PI(3)P in the Rab5^+^ sorting endosome activates Rab11.
• The Rab11^+^ vesicle detaches from the Rab5^+^ sorting endosome, enriches in PI(4)P and traffics to the endocytic recycling compartment.
• PI(4)P in the endocytic recycling compartment recruits the exocyst complex for re-delivery of recycling vesicles to the plasma membrane.
• Phosphatidylserine in the endocytic recycling compartment recruits EDH1, which induces vesicle scission from Rab11^+^ endocytic recycling compartment.
• PI(4)P, phosphatidylserine enriched vesicles can then be re-delivered to the plasma membrane.
• Flotillins may maintain phosphatidylserine-enriched microdomains from the plasma membrane and throughout the endocytic recycling network, facilitating cargo recycling.

### Sorting to the APPL Endosome – A Special Case for Signaling

The APPL^+^ endosome represents a specialized pre-Rab5 endosomal compartment with a unique membrane composition highly enriched in PI that supports continued signaling from receptors internalized by either clathrin-dependent or -independent endocytosis (Lee et al., 2011; Kalaidzidis et al., 2015; Masters et al., 2017; Sposini et al., 2017; Lakoduk et al., 2019). The transition from the endocytic pit to APPL^+^ endosomes is driven by the conversion of PI(4,5)P_2_ to PI by OCRL and INPP5B. Under specific signaling conditions, the 3-phosphatase MTMR2 specifically associates with APPL and, in conjunction with OCRL, can also generate further PI from PI(3,4)P_2_ (Erdmann et al., 2007; Franklin et al., 2013). The APPL^+^ endosome is initially completely devoid of PI(3)P. The substitution of APPL for Rab5 is promoted by acquisition of PI(3)P (Zoncu et al., 2009; Kalaidzidis et al., 2015). Receptors entering the APPL endosome can either be rapidly recycled to the plasma membrane following signaling, or proceed to the Rab5^+^ sorting endosome (Kalaidzidis et al., 2015; Sposini et al., 2017). Crucially, receptors that typically traffic through alternate pathways, such as the EGF receptor, are re-routed to transit the APPL endosome when additional signaling or rapid recycling is required (Lakoduk et al., 2019). The APPL^+^ endosome therefore represents a separate endosomal population to those of cargoes directly entering the Rab5 to Rab7 or Rab11 trafficking pathways with a unique membrane composition that supports further receptor signaling following endocytosis.

### Membrane Heterogeneity Regulates Targeted Trafficking in Polarized Cells

Membrane heterogeneity also controls sorting along specialized endocytic pathways utilized in cell types with distinct polarized apical and basolateral membranes such as hepatocytes, pancreatic cells and kidney epithelia, and in neurons.

Apical and basolateral membranes in polarized cells both contain PI(4,5)P_2_ (Szalinski et al., 2013), while distribution of other phosphoinositides is polarized. PI(3,4)P_2_ is enriched in apical membranes, and PI(3,4)P_2_ is enriched in basolateral membranes (Román-Fernández et al., 2018). Endocytosis is distinct in apical and basolateral membranes in polarized renal epithelia and hepatocytes (Tuma et al., 1999; Szalinski et al., 2013), with cargoes remaining distinctly separated following internalization, even if targeting the same endocytic compartment (Cerneus and Van Der Ende, 1991). As enrichment in PI(3,4)P_2_ facilitates fast endophilin mediated endocytosis (Boucrot et al., 2015; Chan Wah Hak et al., 2018) and PI(3,4,5)P_3_ and PI(4,5)P_2_ facilitates CLIC/GEEC endocytosis (Suetsugu et al., 2006; Lundmark et al., 2008), we speculate that differences in phosphoinositide content in polarized membranes may facilitate different modes of endocytosis. Following endocytosis, rapid segregation of Rab7-targetted and Rab11-targetted recycling cargoes into EEA1-positive or negative endosomes occurs as in non-polarized cells (Leung et al., 2000).

Similarly, sorting in polarized cells also involves maintenance of specific phosphoinositides on endocytic membrane. Inhibition of PI(3)P synthesis in hepatocytes leads to accumulation of apical plasma membrane proteins into large vacuolar structures following endocytosis (Tuma et al., 1999). At the level of recycling endosomes, Rab11^+^ compartments receive cargoes both directly from apical and basolateral endocytosis (Thompson et al., 2007). The role of membrane heterogeneity in regulating the passage of cargoes through Rab11^+^ endosomes in polarized cells is again similar to what we have described for non-polarized cells. Rab11^+^ endosomes contain PI(4)P and phosphatidylserine in kidney epithelia but little PI(3)P (Gagescu et al., 2000; Román-Fernández et al., 2018) and PI(3)P is required for cargo entry into Rab11^+^ transcytosis pathways (Hansen et al., 1995). Transcytosis from the Rab11^+^ recycling endosomal compartment involves the phosphatidylserine binding Rab11-FIP5 protein (Su et al., 2010). Finally, delivery of recycling vesicles to polarized membranes requires PI(4)P enrichment on vesicular membranes, analogous to non-polarized cells (Nguyen et al., 2019).

Neurons use a specialized form of endocytosis called “activity-dependent bulk endocytosis” for rapid retrieval and recycling of synaptic membrane during sustained neurotransmission (Watanabe and Boucrot, 2017). Activity-dependent bulk endocytosis is initiated on PI(4,5)P_2_-enriched membranes, similar to clathrin-dependent endocytosis, but utilizing the BAR-domain containing proteins syndapin and endophilin (Clayton and Cousin, 2009). However, it likely that membrane heterogeneity involving phosphoinositide species contribute to provide specificity to this mode of endocytosis.

Following internalization of cargoes, neuronal trafficking and sorting mirrors that of non-polarized cells indicating that membrane heterogeneity has a similar important regulatory role in neuronal sorting. PI(3)P is required for cargo sorting through Rab5^+^ to Rab7^+^ endosomes and the lysosome (Morel et al., 2013). Loss of WDR91, a protein required for neuronal development, results in accumulation of PI(3)P on endosomes and defective trafficking of cargoes into Rab7^+^ endosomes and lysosomes (Liu et al., 2017). Retromer is recruited to Rab7^+^ endosomes in neurons analogous to non-polarized cells, utilizing the PI(3)P binding VPS29 and is required for regeneration of synaptic vesicles (Singla et al., 2019; Ye et al., 2020). Rab11^+^ endosomes regulates recycling from activity-dependent bulk endosomes (Kokotos et al., 2018) and are highly enriched in phosphatidylserine (Calderon and Kim, 2008), indicating that the distinct membrane domains that segregate Rab11-dependent recycling cargoes from Rab5-to-Rab7 cargoes are similar between neuronal and non-polarized cells.

## Integration of Endocytosis Into Endosomal Subpopulations for Cargo Sorting

The multiple modes of endocytosis that exist in the cell ultimately result in cargoes being integrated into degradative or recycling pathways (Figures 2, 3). As discussed above, we speculate that this cargo fate decision is made prior to cargo entry into the endosomal subpopulations that make up the endocytic sorting pathways. The EGF receptor (EGFR), LDL receptor (LDLR) and G-coupled protein receptors (GPCRs) provide broad examples of how the fate of endocytic cargoes fate is determined prior to or during endocytosis and how this eventually conditions the endosomal subpopulation the cargo enters. Interestingly, this cargo fate decision integrates binding of accessory proteins such as ligands or circulating proteins to the receptor cargo with membrane heterogeneity.

EGFR endocytosis is incredibly complex and is therefore still relatively uncharacterized despite decades of intense research. EGFR is recycled to the plasma membrane or degraded in the lysosome dependent on the concentration of EGF present. At low to medium EGF concentrations, EGFR is internalized by clathrin-dependent endocytosis into multiple potential endosomal subpopulations: AAPL^+^ endosomes for continued signaling and subsequent recycling, autophagosomes for recycling and EEA1^+^ endosomes for degradation (Leonard et al., 2008; Flores-Rodriguez et al., 2015; Fraser et al., 2019; Lakoduk et al., 2019). At high EGF concentrations or inhibition of clathrin, EGF is taken up independently of clathrin *via* macropinocytosis and delivered to the lysosome for degradation, ensuring signaling is terminated (Ménard et al., 2018). It is important to note that while integration of EGFR into these endosomal subpopulations is broadly EGF concentration dependent, these pathways are unlikely to be mutually exclusive.

Membrane heterogeneity is central to the pathway directing EGFR fate. EGFR can be internalized into the cell *via* clathrin pits of differing compositions, inducing recycling or degradation. In clathrin pits containing AP2, EGFR is recycled whereas in the absence of AP2 it is degraded (Pascolutti et al., 2019). Membrane heterogeneity further regulates clustering of EGFR, which in turn results in differing endocytic sorting fates. At low EGF concentrations, the receptor is in small to non-existent clusters, while at high concentrations the receptor exists in large clusters (Bag et al., 2015). Plasma membrane cholesterol is central to EGF receptor cluster size, and potentially the recruitment of Eps15 to the clathrin coated pits required for EGF receptor recycling (Bag et al., 2015). Phosphatidylserine is responsible for maintaining cholesterol in the cytosolic leaflet of the plasma membrane (Maekawa and Fairn, 2015), and Eps15 and its binding partners involved in interaction with AP2, SGIP1α, and FCHO1 all interact with both phosphatidylserine and PI(4,5)P_2_ (Uezu et al., 2007; Hollopeter et al., 2014; Wang et al., 2016). Thus, small EGFR clusters at low EGF concentrations are present in phosphatidylserine enriched regions of the plasma membrane that are primed for delivery to the Rab11^+^ recycling compartment. Macropinocytosis has its own unique phosphoinositide membrane composition (Egami et al., 2014; Maekawa et al., 2014). EGF stimulates the conversion of PI(4,5)P_2_ to PI(3,4,5)P_3_ (Araki et al., 2007) following which PI(3,4,5)P_3_ is sequentially metabolized to PI(3,4)P_2_, PI(3)P and then PI, with each intermediate phosphoinositide recruiting effectors required for the macropinocytic process (Maekawa et al., 2014). Following uptake by macropinocytosis, EGFR is delivered to the lysosome for degradation (Ménard et al., 2018). Membrane heterogeneity therefore is responsible for delivery of EGFR into the endosomal subpopulations required for its recycling or degradation.

LDLR is exclusively internalized by clathrin-dependent endocytosis in non-atherogenic conditions, following which it can be degraded in the lysosome or recycled via the CCC complex (Bartuzi et al., 2016). In contrast to EGFR, LDLR fate is determined primarily by binding of the circulating protein PCSK9 prior to endocytosis. If present in circulation, PCSK9 binds LDLR at the plasma membrane before it is internalized *via* clathrin mediated endocytosis. When bound to PCSK9, LDLR is cleaved by proteases in the cytoplasmic domain (Tveten et al., 2013), which prevents its recovery from Rab7^+^ endosomes by the CCC complex for recycling (Bartuzi et al., 2016; Fedoseienko et al., 2018). Therefore, internalized LDLR likely enters a relatively homogenous subpopulation of endosomes, which is determined by PCSK9 binding at the plasma membrane.

GPCRs are well characterized as undergoing clathrin-dependent endocytosis, following which endosomal signaling occurs and the receptors are recycled to the plasma membrane. Interestingly, while the β2-adrenergic receptor (β2AR) and luteinising hormone receptor (LHR) are both internalized by β-arrestin containing clathrin pits, they immediately enter distinct endosomal subpopulations with different recycling rates (Jean-Alphonse et al., 2014; Sposini et al., 2017). β2AR enters APPL^–^ endosomes, from where relatively slower Rab11-dependent recycling occurs (Puthenveedu et al., 2010; Sposini et al., 2017). By contrast, LHR enters APPL1^+^ endosomes immediately following endocytosis from where rapid return to the plasma membrane occurs (Jean-Alphonse et al., 2014; Sposini et al., 2017). Crucially, signal sequences in the cytoplasmic tails of the receptors dictate the divergent transit of β2AR and LHR into APPL1^+^ compartments (Jean-Alphonse et al., 2014). Interestingly, β2AR resides on two distinct membrane domains on vesicles depending on its state of phosphorylation. Non-phosphorylated β2AR resides in “bulk recycling” membrane domains (Vistein and Puthenveedu, 2013; Bowman et al., 2016), from where actin-dependent, Rab11-dependant recycling to the plasma membrane can occur (Puthenveedu et al., 2010). Phosphorylated β2AR recruits the retromer sorting-nexin SNX1 (Bowman et al., 2016) and SNX27 on EEA1^+^ endosomes (Temkin et al., 2011), prior to the typical retromer interaction on Rab7^+^ endosomes. Together, these results indicate that in special cases cargoes themselves may modify endosome membrane heterogeneity within a single endosomal subpopulation, enabling modulation of cargo recycling rates in specific circumstances to tune cellular signaling outcomes.

## A Unifying Model for Membrane Heterogeneity Regulating Cargo Sorting

The above evidence leads us to present a model for endosomal cargo sorting in which membrane heterogeneity plays a central regulatory role. Decisions on the fate of cargoes are made at the level of endocytic pit formation or rapidly following endocytosis. Cargoes traffic to specific pre-Rab5 endosomes with distinct membrane compositions: (a) EEA1^+^ endosomes highly enriched in PI(3)P for trafficking to the Rab7^+^ late endosome, (b) recycling Rab11^+^ endosomes with high phosphoinositide and phosphatidylserine content and (c) APPL^+^ endosomes highly enriched in PI for sustained endosomal signaling. Local membrane composition within each of these compartments immediately following endocytosis plays a central role in recruiting the membrane tubulating proteins, phosphatases, kinases and Rab proteins required to orientate and maintain cargoes in their respective trafficking pathways.

**FIGURE 4:**
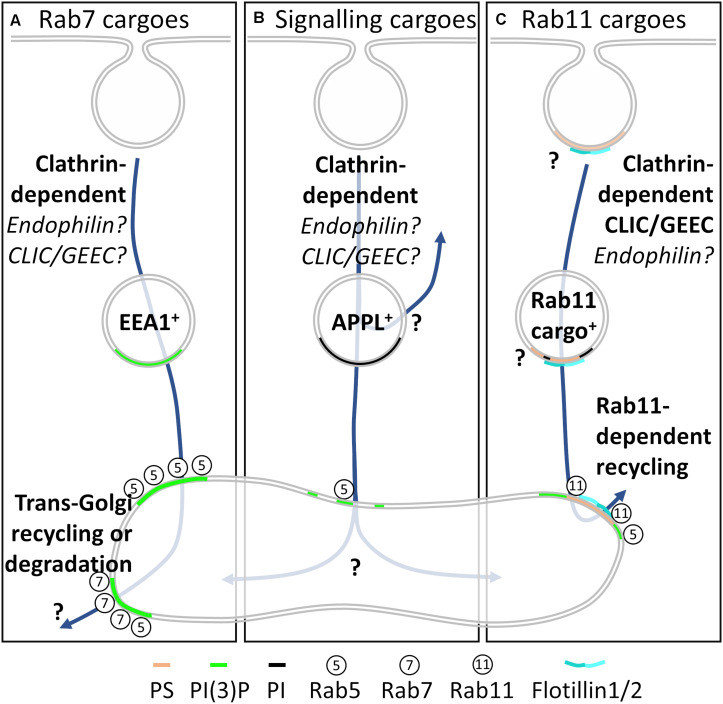
Our model for membrane heterogeneity in endocytic sorting. **(A)** Cargoes destined for *trans*-Golgi recycling or lysosomal degradation enter the cells via clathrin-dependent endocytosis-although fast endophilin-mediated endocytosis and CLIC/GEEC endocytosis may play a role. Endocytic vesicles containing these cargoes acquired EEA1 and are enriched in PI(3)P prior to fusion with the Rab5^+^ sorting endosome. The microdomains containing these cargoes then mature to Rab7^+^ endosomes, from where *trans*-Golgi recycling or degradation can occur. **(B)** Cargoes (receptors) from which signaling is still required following endocytosis are internalized into APPL^+^ endocytic compartments that are enriched in phosphoinositide. Again, fast endophilin-mediated endocytosis and CLIC/GEEC endocytosis may also play roles in cargo delivery. Following signaling from APPL^+^ compartments, cargoes integrate with the Rab5^+^ sorting endosome, from where their fate is not well documented. **(C)** Cargoes destined for Rab11-dependent recycling are internalized from phosphatidylserine-enriched regions of the plasma membrane by both clathrin-dependent endocytosis and CLIC/GEEC endocytosis. Fast endophilin-mediated endocytosis may also play a role, and the range of phosphoinositide compositions of this compartment are not yet established. The flotillin membrane organizing protein family organizes the membrane domains on these phosphatidylserine-enriched endosomes, ensuring segregation throughout the sorting pathway.

In this model, cargoes destined for the late endosomal pathway enter PI(3)P-enriched, EEA1^+^ endosomes (Figure 4A). EEA1^+^ recruits Rab5, generating further PI(3)P. This enriched PI(3)P inactivates Rab5 and recruits Rab7. The cargo can now recycle via retromer/retriever/CCC *trans*-Golgi recycling or be degraded in the lysosome. Rab11-dependent recycling cargoes enter the cell into phosphoinositide and phosphatidylserine-enriched endosomes (Figure 4C). These endosomes destined for Rab11 recycling fuse with the Rab5^+^ sorting endosome, where highly spatially restricted generation of PI(3)P occurs, which is then responsible for recruiting Rab11 and sending cargo to the endocytic recycling compartment. Crucially, phosphatidylserine is maintained throughout this cycle. Cargoes that require further signaling following endocytosis are internalized into PI-enriched APPL^+^ endosomes (Figure 4B). The phosphoinositide enrichment prevents recruitment of Rab5, generating a stable platform from which endosomal signaling can occur. Following signaling from APPL^+^ endosomes, subdomains of the endosome can acquire Rab5 for cargo entry into the Rab5^+^ sorting endosome or be recycled directly to the plasma membrane.

For each of these endosomes, the membrane composition immediately following endocytosis controls all further downstream acquisition of proteins and membrane constituents that ultimately control the fate of a cargo. Cargo fate decisions are therefore made at the earliest stages of endocytosis, with membrane heterogeneity enforcing this decision.

## Remaining Questions and Conclusion

Membrane heterogeneity is a central regulator of cargo endocytosis and sorting. At the center of each mode of endocytosis, a specific membrane environment ensures that endocytosis occurs at a specific time and place within the cell. Immediately following endocytosis, cargoes are segregated into endosomes with specific membrane compositions that dictate whether they ultimately sort into Rab7^+^ endosomes for recycling or degradation, or Rab11^+^ endosomes for recycling.

While it is clear membrane heterogeneity is a crucial regulator at each step of the endocytosis and sorting process, questions remain (Figure 4). How can heterogenous membrane domains in the same endocytic mode sort for different fates at the level of the plasma membrane? What fates can cargoes endocytosed by clathrin-independent pathways be sorted into, and how does membrane heterogeneity contribute to this? What other membrane lipid species are involved in regulating the endocytic sorting process? Given the nuanced differences in endocytic sorting between polarized and non-polarized cells, do membrane lipids play the same role in polarized trafficking? Do specialized endocytic pathways such as activity-dependent bulk endocytosis and transcytosis segregate cargo utilizing similar lipid microdomains or do specialized domains exist for delivery to specific polarized membranes?

Investigation of the lipid species contributing to membrane heterogeneity have typically relied on biochemical or pharmacological approaches [e.g., cholesterol depletion by methyl-β-cyclodextrin (Chadda et al., 2007), PI(3) kinase inhibition (Boucrot et al., 2015)]; genetic approaches [e.g., PIK3C2α knockout (Franco et al., 2014)]; fixed and live cell imaging studies using fluorescent lipid markers (Ketel et al., 2016) and constitutively active/dominant negative Rab proteins (Liu et al., 2017). While these have provided incredibly useful information on the role of membrane heterogeneity in cellular trafficking, each has limitations. Biochemical and pharmacological approaches carry inevitable off target effects, which have recently been highlighted with methyl-β-cyclodextrin (Hirama and Fairn, 2018). Knockdowns, knockouts and overexpression of constitutively active/dominant negative proteins all have potential unwanted effects. Because of the relatively long-term nature of the protein disruption, compensatory mechanisms can be activated potentially obscuring the results (El-Brolosy and Stainier, 2017). We believe transient optogenetic manipulation of membrane lipids and proteins offers a specific, minimally disruptive method to overcome the majority of the issues observed with biochemical/pharmacological and knockdown/knockout approaches (detailed below).

Genetically encoded biosensors have greatly enhanced our ability visualize the lipid species contributing to plasma membrane and endosomal membrane heterogeneity (Wills et al., 2018). To fully dissect the roles of membrane heterogeneity in endosomal sorting, the generation of biosensors to further lipids enriched in membrane domains will allow a better understanding of their role in endocytosis and trafficking. Further, developing new lipid biosensors that have differing affinities for the same lipid will allow observation of a wider range of cellular events [excellently detailed in Wills et al. (2018)]. Development of nuanced biosensors able to detect protein activation will also provide further advances, with the recent Rab11 biosensor (Campa et al., 2018) providing interesting insights into the role of PI(3)P in the Rab11 recycling pathway.

Advances in light and electron microscopy techniques coupled with lipid biosensors will provide the next leap in understanding of how membrane heterogeneity controls endocytic processes. Optogenetic metabolisers that couple a phosphoinositide phosphatase or kinase to plasma membrane anchors have proven to be extremely effective tools for dissecting functions of various phosphoinositides (Idevall-Hagren et al., 2012; Kakumoto and Nakata, 2013). Further iterations of optogenetic systems have coupled a specific optogenetic metaboliser to a specific subcellular compartment, allowing precise dissection of how a membrane species functions on a single endosomal population, as has been recently performed to investigate the role of PI(4)P on Rab3^+^ vesicles (Nguyen et al., 2019). Extension of current optogenetic lipid metabolisers to control of other phosphoinositides and membrane species such as cholesterol and phosphatidylserine will allow us to determine precisely their roles in endocytosis and sorting.

Live total internal fluorescence microscopy (TIRF) has provided striking advances of our knowledge of the function plasma membrane lipid species (Ketel et al., 2016). Advances in live cell imaging methodologies beyond TIRF will further advance our understanding of membrane heterogeneity in intracellular populations. We have recently generated methods utilizing single- and two-photon photoactivation of fluorescent proteins to elucidate every step of the T cell receptor endocytic sorting process (Compeer et al., 2018; Redpath et al., 2019). Further, we coupled cargo photoactivation to optogenetic inhibition of Rab proteins (Redpath et al., 2019). Photoactivation of cargo proteins coupled with lipid biosensors, photoactivation of lipid biosensors themselves and coupling of cargo photoactivation with optogenetic activation or inhibition of lipid species, followed by tracking throughout their endocytic routes will undoubtedly advance our knowledge on their role in endocytic sorting.

Finally, this review has focussed on a subset of endocytic processes and endosomal pathways. Each endocytic pathway will have its own unique composition of membrane domains that regulates function, as is the case for clathrin-dependent endocytosis, fast endophilin-mediated endocytosis and CLIC/GEEC endocytosis, and other pathways such as macropinocytosis (Kerr and Teasdale, 2009). Similarly, other endosomal sorting pathways such as polarized apical and basolateral recycling pathways, transcytosis and synaptic vesicle recycling will have their own endosomal membrane heterogeneity that regulates cargo fate. Further investigation and characterization of the membrane compositions of these endocytic pathways will greatly further our understanding of how cargo trafficking is controlled in the cell.

## Author Contributions

GR and JR conceived of and wrote the manuscript. GR, VB, PR, and JR prepared the figures. VB and PR provided critical editing and input. All authors contributed to the article and approved the submitted version.

## Conflict of Interest

The authors declare that the research was conducted in the absence of any commercial or financial relationships that could be construed as a potential conflict of interest.
